# Report of a Case of Strangulated Hernia

**Published:** 1897-11

**Authors:** W. W. Walker

**Affiliations:** Schulenburg, Texas


For the Texas Medical Journal.

### REPORT OF A CASE OF STRANGULATED HERNIA.

BY W. W. WALKER, M. D., SCHULENBURG, TEXAS.

JUNE 28th, 1897, I was called by telephone to Weimar to operate on a case of strangulated hernia. Patient, F. E., a young married man; æt about 30 years. He is a saloon man; had been drinking pretty hard for several years; very much bloated, with an enormously enlarged liver. When I arrived, about 10 p. m., Drs. Pothast and Murchison, his physicians, told us that Mr. E. had just returned from San Antonio the day before. While there a very prominent surgeon had examined him, and made an incision into his scrotum, and then told him he had better go home, he had dropsy of the heart, and was in no condition to stand an operation. Dr. Pothast had aspirated twice, and handed me a bottle containing about six ounces of bloody serum. The abrupt termination of swelling at the abdominal wall indicated hydrocele or hematotele, the vomiting and constipation, with a knowledge of an old hernia, made the diagnosis positive. Mr. E. was in an extremely bad condition for any operation, the general anasarca, enlarged liver, faulty heart action, the bistoury incision and wounds from aspirator needle, made the prognosis very grave, but there was no alternative except operation, and take chances. Mrs. Walker administered anesthetic, Drs. Pothast and Murchison assisting me. After cutting down to and opening the sack, I found the gut had not come down through the internal abdominal ring, but was through the abdominal wall direct, with very small opening. The internal ring, with cord and vessels, was perfectly intact. This accounted for the abrupt termination, and made the tumor feel like a hematocele, and might account for an error of diagnosis, and the use of a bistoury in San Antonio. The opening was enlarged with a hernia knife, the bowel, which was very black, and in an almost sloughing condition, was returned and held in place by Dr. Pothast while the sack was cleansed out, removing one old clot of offensive black blood probably three inches long, one and one-half inches broad, and one-half inch thick. The abdominal opening with peritoneum was closed by three silk sutures cut short, the wound was thoroughly washed out, disinfected, dusted with iodoform, and closed with silk. Reaction was good. The patient was given one grain each of calomel and soda every hour until ten were taken; also ammonia, strychnia and digitalis. Coming home that night, I told my wife Mr. E. would be my first death from hernia operation, and I would not give twenty-five cents for his chances. I saw him again on the 30th with Drs. Pothast and Murchison. His bowels had moved freely, heart's action was good, the wound had not suppurated, and all indications pointed to a speedy recovery. I had hardly reached home when a terrible attack of delirium tremens came on, which taxed the patience and ingenuity of his physicians. However, they got him through, and all was lovely until July 8th, when I was called for one of those unfortunate things that will bob up in spite of our best laid plans. This was the cumulative action of digitalis. His heart was bounding and raising the chest wall like a trip-hammer. Digitalis was discontinued, and he was placed on large doses of ammonia, mur. strychnia and tinct. cinchona com. and he made a good recovery.

Mr. E. is now attending to his business, and I hope a happier and a wiser man than to take another tussel with John Barley-corn.

